# Modification and application of “zero-line” incision design in total endoscopic gasless unilateral axillary approach thyroidectomy: A preliminary report

**DOI:** 10.3389/fsurg.2023.1121292

**Published:** 2023-02-23

**Authors:** Huiling Wang, Rui Liu, Chaojie Zhang, Qian Fang, Zheng Zeng, Wanlin Wang, Shuo You, Meng Fang, Jinhao Dingtian

**Affiliations:** Department of Breast and Thyroid Surgery, Hunan Provincial People's Hospital/The First Affiliated Hospital of Hunan Normal University, Changsha, China

**Keywords:** gasless endoscopic thyroidectomy, surgery, thyroid cancer, lymphadectomy, incision activity

## Abstract

**Introduction:**

Gasless unilateral trans-axillary approach (GUA) thyroidectomy has witnessed rapid development in technologies and applications. However, the existence of surgical retractors and limited space would increase the difficulty of guaranteeing the visual field and disturb safe surgical manipulation. We aimed to develop a novel zero-line method for incision design to access optimal surgical manipulation and outcomes.

**Methods:**

A total of 217 patients with thyroid cancer who underwent GUA were enrolled in the study. Patients were randomly classified into two groups (classical incision and zero-line incision), and their operative data were collected and reviewed.

**Results:**

216 enrolled patients underwent and completed GUA; among them, 111 patients were classified into the classical group, and 105 patients were classified into the zero-line group, respectively. Demographic data, including age, gender, and the primary tumor side, were similar between the two groups. The duration of surgery in the classical group was longer (2.66 ± 0.68 h) than in the zero-line group (1.40 ± 0.47 h) (*p* < 0.001). The counts of central compartment lymph node dissection were higher in the zero-line group (5.03 ± 3.02 nodes) than that in the classical group (3.05 ± 2.68 nodes) (*p* < 0.001). The score of postoperative neck pain was lower in the zero-line group (1.0 ± 0.36) than that in the classical group (3.3 ± 0.54) (*p* < 0.05). The difference in cosmetic achievement was not statistically significant (*p* > 0.05).

**Conclusion:**

The “zero-line” method for GUA surgery incision design was simple but effective for GUA surgery manipulation and worth promoting.

## Introduction

Endoscopic or minimal invasive thyroid surgery has rapidly spread in recent decades (1–3). Currently, the commonly applied techniques according to surgical approaches could be divided into trans-thoracic, trans-axillary, trans-oral, trans-cervical, and axillo-breast approaches (4). In Asian centers, especially in China, GUA has been widely applicated and accepted since modified and improved in 2017 by Ge et al. (5). Compared with others, the gasless unilateral axillary approach (GUA) can establish a surgical space without carbon dioxide (CO2) insufflation and gas-related complications such as gas embolism and acidosis (6). Furthermore, the learning curve is relatively short for skilled surgeons to obtain, and surgical efficacy and cosmetic outcomes are as well as other techniques (7–9). However, the surgical space separation was achieved with a dedicated surgical retractor, which, together with the limited operating space, would increase the difficulty of guaranteeing the visual field and disturb the surgeon's manipulation. In addition, dissection of inferior thyroid areas, including recurrent laryngeal nerve and the inferior border of the VI area neck lymph node compartment, could be interfered with by the clavicular head of the sternocleidomastoid muscle. Hence, we modified the design of the incision approach and called it the “zero-line” method. Also, we compared the effects of zero-line incision and the classical incision approach in surgical outcomes.

## Materials and methods

### Inclusion and exclusion criteria

Patients who underwent GUA surgery in our hospital from October 2021 to August 2022 were included. All patients were diagnosed with thyroid cancer by fine needle aspiration (FNA) pathological examination and/or BRAF V600E before surgery. The further enrollment criteria were as below (10–12): first, the diameter of thyroid nodules was smaller than 2 cm; second, all lesions are unilateral, and intra-lobe, and the contralateral thyroid lobe could be preserved; third, no clinical involved cervical lymph nodes (cN0) was detected by imaging examination preoperatively; last, no obvious abnormality in coagulation, cardiopulmonary, liver, and kidney function preoperatively. The 217 patients were randomly classified into two groups (classical design or zero-line design). This research was approved by the Human Research Ethics Committee (No.1280) at the Hunan Provincial People's Hospital. All patients had completed the GUA operation by the same group of surgeons with informed consent obtained.

### Surgical procedures

**Figure 1:**
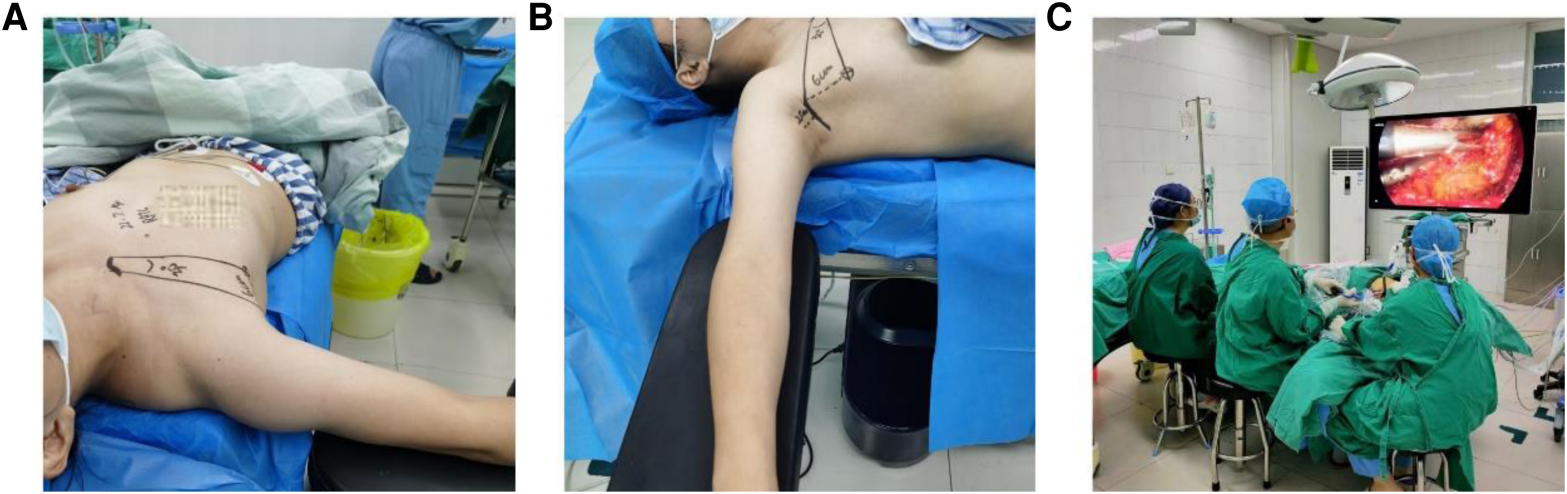
Patient's posture and surgeon's seat. (**A**) Place the operated side body close to the edge of the surgical bed. (**B**) The arm was abducted at 90°. (**C**) The HD monitor was placed contralateral, and the surgeon and assistant were then seated on either side of the patient's arm.

After generally anesthetized under tracheal incubation, the patient was placed in a supine position on a pad positioner, with the neck gently extended using a mildly sloping pillow under the shoulder and neck. Place the operated side body close to the edge of the surgical bed (Figure 1A), and the arm was naturally abducted at about 90 degrees at the arm board (Figure 1B), which could be adjusted if the clavicle is higher than the thyroid isthmus. The monitor was placed contralateral, and the surgeon and assistant were seated on either side of the patient's arm (Figure 1C).

**Figure 2:**
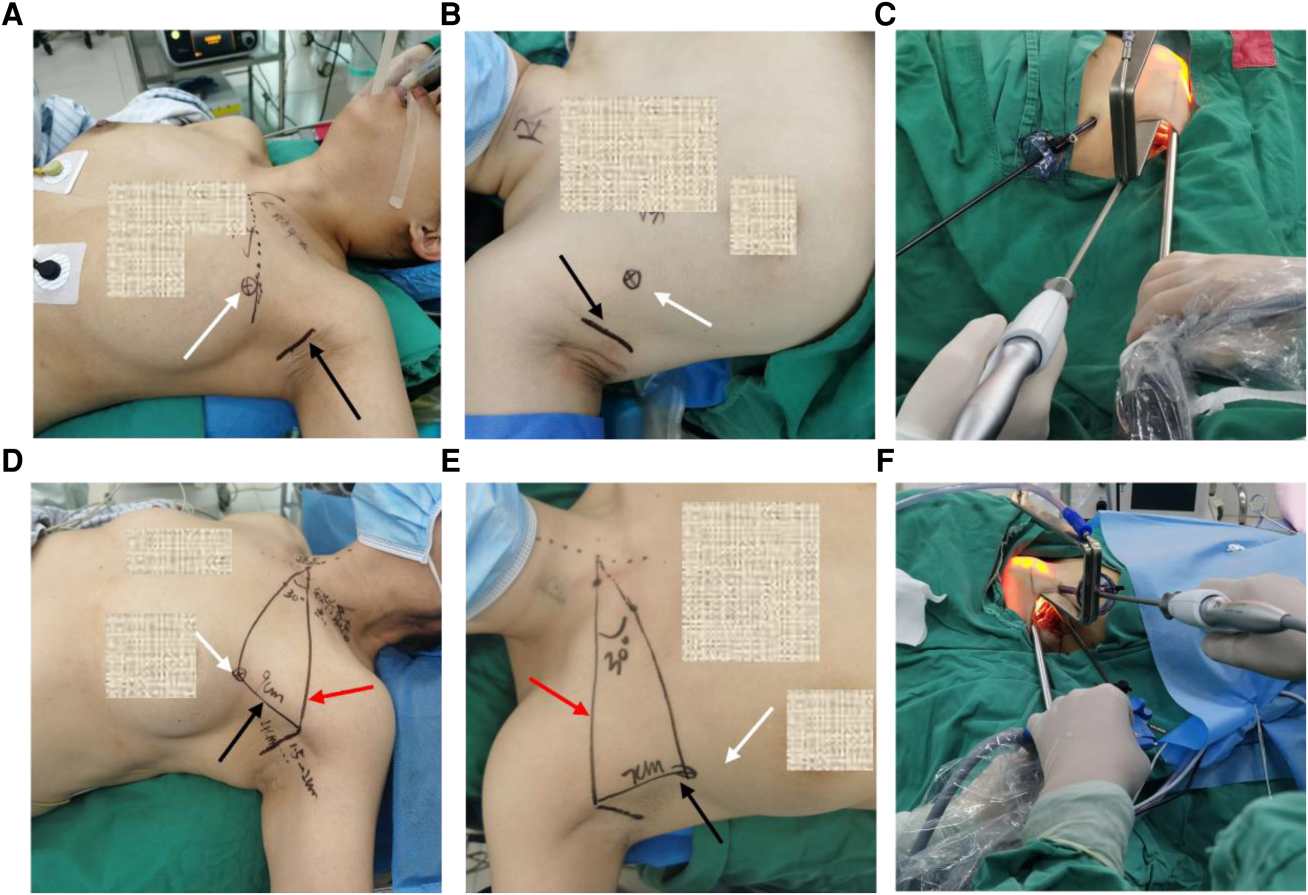
Representative photos of zero-line and classical incision design. (**A,B**) Classical designation on body surface for left-side and right-side thyroidectomy. (**C**) Endoscope, surgical clamp, and auxiliary clamp cooperation for left-sided thyroidectomy. (**D,E**) Zero-line designation on body surface for left-side and right-side thyroidectomy. (**F**) Endoscope, surgical clamp, and auxiliary clamp cooperation for right-sided thyroidectomy. Black row indicated the main incision, white row indicated the trocar incision, red row indicated the zero-line.

For the classical design (5), the main oblique incision (about 3.5–4.5 cm in length) was made along the armpit's first or second natural skin fold. It should not exceed the anterior axillary line, whereby the endoscope and surgical instrument were placed. In addition, we made a 0.5 cm small incision at the intersection of the axillary front line and the upper edge of the breast; the location was 3.0–4.0 cm underneath the main incision, whereby a 5 mm trocar, and the cannula was then inserted (Figures 2A,B). For the zero-line design, an oblique incision (about 3.5–4.5 cm in length) parallel to the armpit stripes was made about 2 cm from the axillary top. The front end should not exceed the anterior axillary line. Define the line connecting the intersection of the incision with the lateral border of the pectoralis major and the highest point of the clavicle as the zero-line. After that, define the intersection of the reverse extension line of zero-line and the anterior midline of the chest (midline of the sternum) as the apex point, then draw a straight line along a 30-degree counterclockwise angle. A 0.5 cm trocar incision is then made at the intersection of this line and the lateral border of the pectoralis major; the 30-degree angle could be slightly different due to right-handed habit. When choosing the site of the trocar incision for a female patient, the breast should be retracted inferiorly, and kept the chest skin flattened (Figures 2D,E).

### Data collection and outcomes

The gender, age, body mass index (BMI), size of largest tumor, primary tumor side, total operation time, intraoperative blood loss, postoperative pathological examination of central lymph nodes, and the score of postoperative neck pain were compared between the two groups of patients. Operative time was defined as the time from the initial skin incision to the point of final closure. Also, the postoperative neck pain was evaluated by the standard pain scoring method 48 h after surgery. Score 5 was defined as an extremely severe inability to complete daily activities. The evaluation of incision cosmetic achievement was evaluated by a visual analog scale after 30 days of leaving the hospital. A score of 0 was defined as extremely dissatisfied, and a score of 10 was defined as well satisfied.

### Statistical analysis

We used SPSS 27.0 software (Armonk, NY: IBM Corp) for statistical analysis. Categorical variables were analyzed using the Chi-squared test. The normality of continuous data was analyzed using the Shapiro-Wilk test. Continuous data were analyzed using an unpaired t-test or Mann-Whitney test. A *p*-value of <0.05 was considered statistically significant.

## Results

### Completion of endoscopic surgery

Between October 2021 and August 2022, 217 enrolled participants diagnosed with thyroid cancer underwent GUA. Of all the 217 patients, one patient of the classical design group was converted to open surgery due to intraoperative bleeding, and the rest 216 patients completed the endoscopic surgery successfully. 111 patients (83 females and 28 males) in the classical design group had an average age of 36.84 ± 11.12 years, and 105 patients (82 females and 23 males) in the zero-line design group had an average age of 38.87 ± 10.95 years, respectively. Demographic data, including age, gender, BMI, size of the largest tumor, and the primary tumor side, were similar between the two groups (Table 1). There was no issue of postoperative bleeding, permanent hypoparathyroidism, recurrent laryngeal nerve or superior laryngeal nerve injury in both groups. In all cases, the scapula hyoid muscle was retained *in situ* successfully.

**Table 1:** General conditions of two groups.

Group (*n*)	Age	Gender	BMI	Size of largest tumor (cm)	Primary tumor side
Classical (111 cases)	36.84 ± 11.12	83 F and 28 M	23.36 ± 3.715	0.93 ± 0.39	42 L and 69 R
Zero-line (105 cases)	38.87 ± 10.95	82 F and 23 M	23.38 ± 3.802	0.89 ± 0.51	49 L and 56 R
*p*	>0.05	>0.05	>0.05	>0.05	>0.05

### Operative duration, lymph node dissection, and bleeding

The operative time in the classical design group was longer than in the zero-line design group, with an average time of 2.66 ± 0.68 h and 1.40 ± 0.47 h (*p* < 0.001). The counts of central compartment lymph node dissection were higher in the zero-line design group (5.03 ± 3.02 nodes) than that in the classical design group (3.05 ± 2.68 nodes) (*p* < 0.001). The difference in the volume of intraoperative bleeding between the two groups was not statistically significant (*p* > 0.05) (Table 2).

**Table 2:** Operative indicators of two groups.

Group (*n*)	Operative duration (hours)	Counts of central lymphnode dissection	Bleeding volume (ml)	Score of postoperative neck pain	Score of cosmetic achievement
Classical (111 cases)	2.66 ± 0.68	3.05 ± 2.68	11.53 ± 7.32	3.3 ± 0.54	9.28 ± 0.41
Zero-line (105 cases)	1.40 ± 0.47	5.03 ± 3.02	10.86 ± 6.98	1.0 ± 0.36	9.55 ± 0.44
*t*	16.33	−5.10	0.69	31.61	−4.60
*p*	<0.001	*p* < 0.001	>0.05	<0.001	>0.05

F, female, M, male, L, left, R, right.

### Postoperative pain and cosmetic satisfaction

The score of postoperative neck pain was lower in the zero-line design group (1.0 ± 0.36) than that in the classical design group (3.3 ± 0.54) (*p* < 0.05). The difference in the visual analog scale of cosmetic achievement was not statistically significant in the two groups (*p* > 0.05) (Table 2). Representative photos of the comparison of recovery of the postoperative surgical incision in two groups were shown in Supplementary Figure S1.

## Discussion

The significant advantages in the GUA approach for unilateral thyroidectomy included the excellent cosmetic effect. Also, the gasless method can effectively avoid CO2-related complications. Also, the technique is relatively easy for surgeons to master and promote, and the curative effect is also equivalent and satisfactory compared with other surgical methods (9, 13–16). Further, recent advances in neuromonitoring and energy-based device also made video-assisted sutureless thyroidectomy safe and effective (17, 18). In the GUA method, surgeons first accessed to the surgical field from the axillary, then dissected and exposed the unilateral thyroid in the space between the clavicular head and the sternal head of the sternocleidomastoid muscle. After that, adequate tension was kept using the retractors, which suspended the unilateral thyroid above the surgical field. Surgeons first separated the posterior of the thyroid, then dissected the thyroid gland from the lateral to the inferior. Therefore, avoiding interference between the forceps, the endoscope, and the retractor during the operation is pivotal. Further, how to avoid the operative blockage of the clavicle head on the lower pole of the thyroid gland, the recurrent laryngeal nerve, and the lower border of the central lymph node compartment remains significant for a successful operation (9, 19). However, the smoothness and difficulty of the operation often suffer due to the unreasonable design of the incision approach.

**Figure 3:**
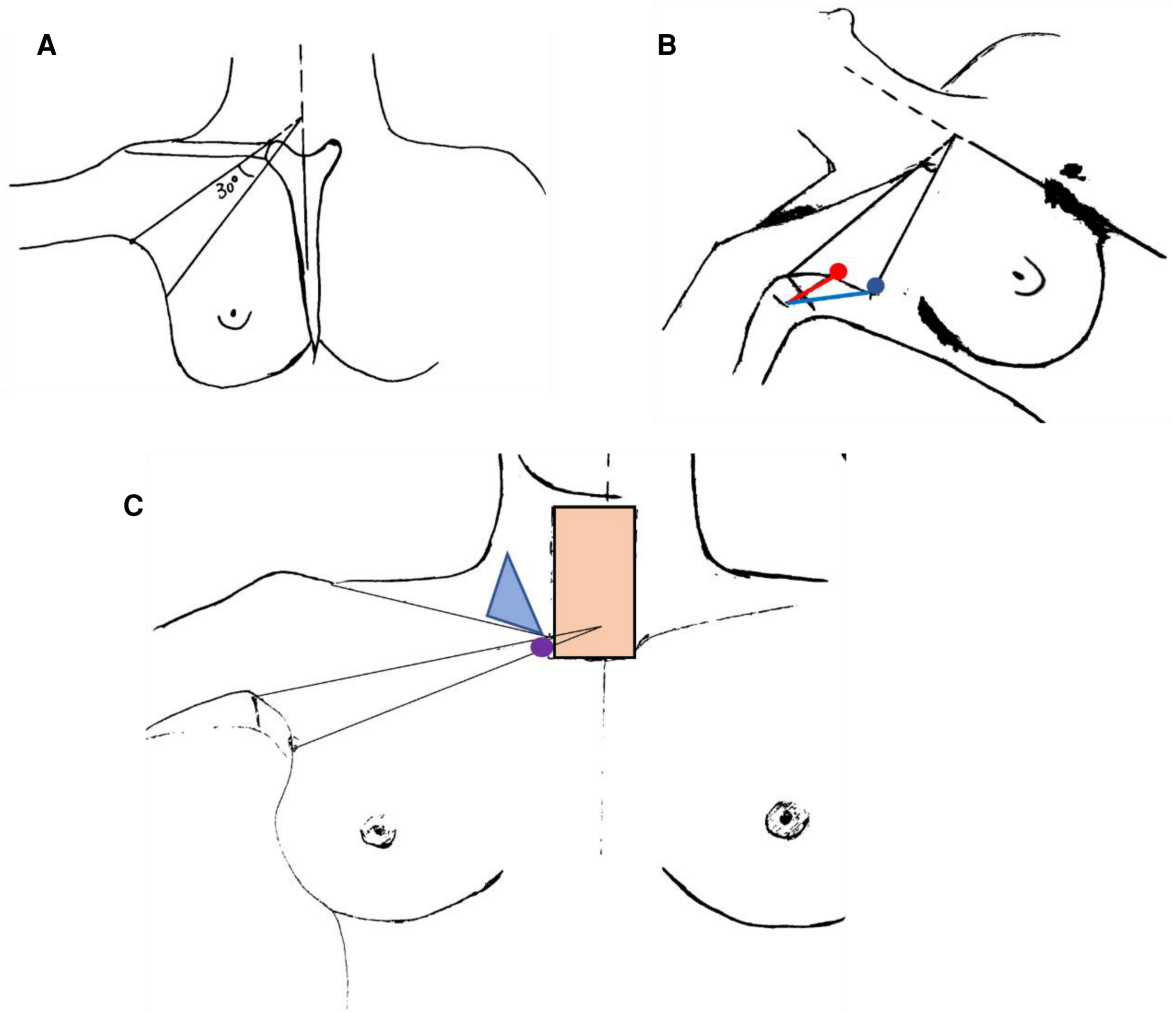
Schematic diagrams of zero-line and classical incision design. (**A**) Schematic diagram of zero-line incision design. (**B**) Comparative diagram of the two designs representing different positions of trocar incision. The red dot and line indicated the classical trocar incision and the distance to the main incision, and the blue dot and line indicated the zero-line ones. (**C**) Diagram representing the lever principle of zero-line incision design. The purple dot indicated the fulcrum (clavicular head of sternocleidomastoid). The light blue triangle indicated the supraclavicular fossa. The meat-colored square indicated the surgical lesion of thyroidectomy.

Our study provides a zero-line incision design method. The method uses relatively fixed markers on the human body, by which the operational space could be granted even with variation from gender, neck length, chest width, and weight status. In our experience, the zero-line method reduced the mutual interference of surgical instruments during the operation. As shown in Figure 3, the scope of the auxiliary clamp's activities was determined by the distance from the auxiliary trocar incision to the main incision. When the distance is longer, the incidence of surgical instruments' mutual interference will be reduced according to the lever principle, which would be the fundamental superiority of zero-line design. In zero-line design, the distance was determined by the location relationship of each anatomy marker and could be flexibly changed in a different patient, which was usually longer than in classical design. While in classical design, a settled distance (3–4 cm) makes the dissecting line relatively far from the superior margin of the mammary gland, and the activity space of the auxiliary clamp is more seriously restricted (9). In short, zero-line design enlarges the scope of surgical instruments' activity and leads to universally smoother operations.

At the same time, the ample operating space by zero-line approach significantly improved the lower thyroid gland separation and the central lymph node dissection. More tissues were dissected in the zero-line design group since the activity scope of the auxiliary clamp was significantly larger than the classical group. In many hospitals, including our center, prophylactic central neck dissection will be applied in patients with thyroid cancer to reduce the possibility of recurrence, and clinical uninvolved lymph nodes (cN0) by imaging examination but with cancer metastases will be diagnosed in postoperative pathological examination (20, 21). In GUA surgery, recognition and protection were usually easily handled for the superior parathyroid gland. Still, the inferior parathyroid gland in both groups was almost undetectable and protected in the endoscopic vision. However, by immediate auto-transplantation combined with unilateral surgery, no contemporary or persistent postoperative hypoparathyroidism in both group. Further, patients experienced less neck discomfort under zero-line design after surgery. We believe the main reason is the shorter operative time of the zero-line group, which leads to a slighter skin flap extraction and suspension in surgical field exposure.

## Conclusion

In conclusion, we modified the “zero-line” method for incision design, and the method was simple but effectively facilitated GUA surgery and was worth promoting. The zero-line incision design enlarges the scope of surgical instruments' activity and leads to smoother operations, slighter skin flap extraction and more radical lymph node dissection. However, there were still limitations to our study. First, the sample size for the zero-line method cases we completed was still insufficient, and we hope that multi-center trials can be conducted and promoted. Second, patients reported their visual cosmetic achievement 30 days after hospitalization, which requires longer follow-ups. Also, we must pay attention to the technical requirements and difficulties of the GUA approach for bilateral thyroid surgery in the future. Last, the learning curve for the surgery should not be ignored.

## Data Availability

The original contributions presented in the study are included in the article/**Supplementary Material**, further inquiries can be directed to the corresponding author/s.
