# Supplementary material for: Modification and application of “zero-line” incision design in total endoscopic gasless unilateral axillary approach thyroidectomy: A preliminary report
Source: Front Surg. 2023 Feb 23;10:1121292. doi: 10.3389/fsurg.2023.1121292 (PMC9995670; doi:10.3389/fsurg.2023.1121292)

Supplementary Figure 1. Representative pictures about the recovery of post-operative surgical incision in two groups. (A) Representative pictures of classical incision design. (B) Representative pictures of zero-line incision design. Up: picture of anterior cervical region. Down: picture of armpit region.
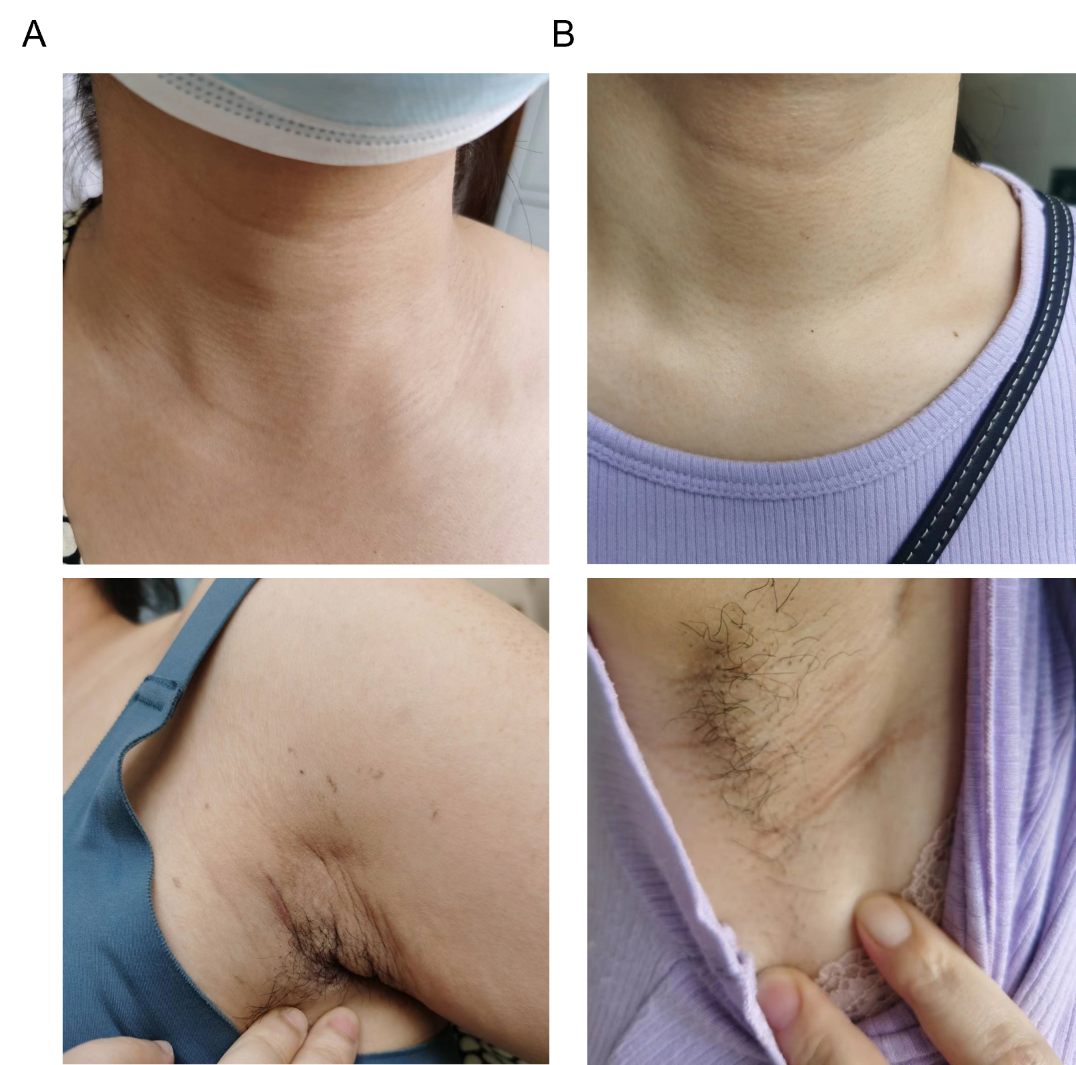

Supplement: Supplementary file 1 [file Datasheet1.docx]
